# Seahorse-assessed metabolic data, CAM assay data and aspect ratio data of tenocyte in vitro cultures treated with insulin-like growth factor-1 (IGF-1), with platelet-derived growth factor-BB (PDGF-BB) or with IGF-1 and PDGF-BB simultaneously

**DOI:** 10.1016/j.dib.2025.111942

**Published:** 2025-07-31

**Authors:** Julia Rieber, Petra Wolint, Gabriella Meier-Bürgisser, Esteban Ongini, Pietro Giovanoli, Maurizio Calcagni, Jess G. Snedeker, Johanna Buschmann

**Affiliations:** a Division of Plastic Surgery and Hand Surgery, University Hospital of Zurich, 8091 Zurich, Switzerland; b Balgrist University Hospital, University of Zurich, 8008 Zurich, Switzerland; c Institute for Biomechanics, ETH Zurich, 8092 Zurich, Switzerland

**Keywords:** Angiogenesis, Metabolism, Tenocytes, Tendon repair

## Abstract

As tendon rupture repair still implies problematic outcomes for the patients, such as re-rupture or adhesion formation to the surrounding tissue, restricting the capability for full loading or the range of motion, novel approaches are welcome and many research groups are working on the optimization of tendon repair. For example, growth factor delivery to the ruptured tendon may support the natural healing by accentuating growth factor dynamics that otherwise occur naturally.

We have therefore tested insulin-like growth factor-1 (IGF-1) and platelet-derived growth factor-BB (PDGF-BB) or both mixed together under in vitro conditions, supplementing the growth factors to cell culture medium of rabbit Achilles tenocytes and rabbit adipose-derived stem cells. We have assessed the effects on the cell morphology and provide aspect ratio data, as well as metabolic data determined by Seahorse experiments, and finally CAM assay data referring to several angiogenic readouts, respectively.

## Graphical abstract

**Figure:**
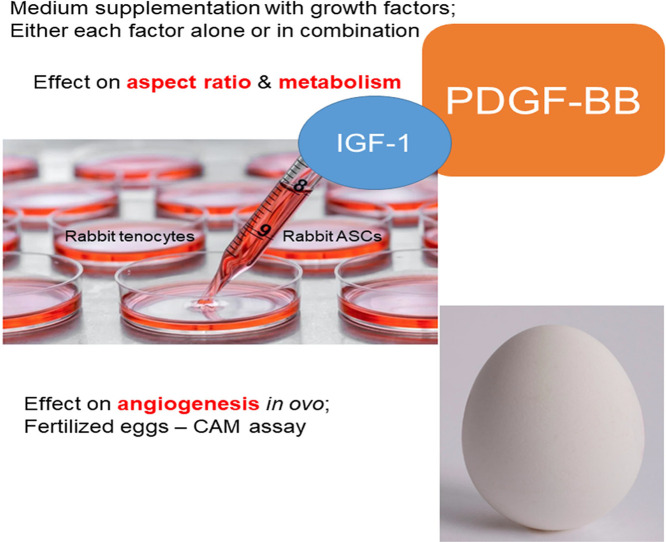

Specifications Table

**Table:** 

Subject	Angiogenesis, cell biology, metabolism
Specific subject area	Cell cultures of rbADSCs or rbTenocytes were supplemented with IGF-1, with PDGF-BB or with both growth factors simultaneously. The aspect ratio of the cells was determined. Furthermore, metabolic data determined with the Seahorse assay are presented. Finally, the CAM assay was performed and angiogenic data are provided.
Data format	The data consist of raw and analysed data.
Type of data	Excel files of the type *.xlsx* files.
Data collection	Data collection was performed with different kinds of experiments, including cell cultures of either rbADSCs or rbTenocytes; to assess the aspect ratio of the cells under growth factor treatment. Moreover, Seahorse experiments were performed to assess the influence of the growth factors on the metabolism and to study potential interactions or synergism of the two growth factors.
Data source location	Except for the Seahorse data, all data were collected in Zurich, Switzerland, at the University Hospital Zurich in the laboratories of the Department for Plastic Surgery and Hand Surgery. The Seahorse measurements were performed at ScopeM, at ETH Zurich, Switzerland.
Data accessibility	**Repository name**: Mendeley Data **Data identification number**: 1. DOI:10.17632/c6p2zpnrgd.2 2. DOI:10.17632/7tk2c47vkf.1 3. DOI:10.17632/kbb4pwmg5g.1 **Direct URL** to data: 1. Aspect ratio data of rabbit Achilles tenocytes in vitro culture treated with Insulin-Like Growth Factor-1 (IGF-1), with Platelet-Derived Growth Factor-BB (PDGF-BB), or with both growth factors - Mendeley Data 2. CAM assay data for chorioallantoic membrane treated with Insulin-Like Growth Factor-1 (IGF-1), with Platelet-Derived Growth Factor-BB (PDGF-BB), or with both growth factors - Mendeley Data 3. Seahorse-assessed metabolic data for rabbit adipose-derived stem cells and rabbit Achilles tenocytes treated with Insulin-Like Growth Factor-1 (IGF-1), with Platelet-Derived Growth Factor-BB (PDGF-BB), or with both growth factors simultaneously - Mendeley Data
Related research article	Synergistic Effects of Insulin-like Growth Factor-1 and Platelet-Derived Growth Factor-BB in Tendon Healing - PubMed

## 1 Value of the Data

• Our data can be reused, if other rabbit cell types than rabbit adipose-derived stem cells or rabbit tenocytes are used to be supplemented with IGF-1 and/or PDGF-BB.
• These data can be reused, if other species than rabbits are used for the extraction of ADSCs or tenocytes.
• These data can be compared with other growth factors than IGF-1 and/or PDGF-BB that are supplemented to the CAM assay.
• Our Seahorse data set can be re-analyzed and re-used to elucidate signaling pathways affecting tendon metabolism.

## 2 Background

Growth factors (GFs) play a pivotal role during tendon healing and differentiation of tendon stem progenitor cells towards the tenogenic phenotype [[1], [2], [3], [4], [5]]. GFs exhibit distinct spatiotemporal dynamics and the fine-tuned balance among GFs is of high importance for a regenerative healing. Among then, early appearing platelet-derived growth factor-BB (PDGF-BB) and later occurring insulin-like growth factor-1 (IGF-1) contribute to proliferation, extracellular matrix synthesis and wound healing [2,6]. In order to circumvent the natural dynamics and instead accentuate and elucidate the proposed synergistic effects of these two GFs, an in-depth study was performed where data for cell metabolism, cellular reorganization and angiogenic response were determined under IGF-1 supplementation, PDGF-BB supplementation or both GFs together in an in vitro rabbit Achilles tenocyte monolayer culture [1].

## 3 DATA Description

The data are stored as a set of Microsoft Excel files (Microsoft Corporation, Redmond, WA, USA) (.xlsx files) in three Mendeley Data repository service entitled (i) Aspect ratio data of rabbit Achilles tenocytes in vitro culture treated with Insulin-Like Growth Factor-1 (IGF-1), with Platelet-Derived Growth Factor-BB (PDGF-BB), or with both growth factors - Mendeley Data; (ii) CAM assay data for chorioallantoic membrane treated with Insulin-Like Growth Factor-1 (IGF-1), with Platelet-Derived Growth Factor-BB (PDGF-BB), or with both growth factors - Mendeley Data; and (iii) Seahorse-assessed metabolic data for rabbit adipose-derived stem cells and rabbit Achilles tenocytes treated with Insulin-Like Growth Factor-1 (IGF-1), with Platelet-Derived Growth Factor-BB (PDGF-BB), or with both growth factors simultaneously - Mendeley Data.

Each of these repositories contains one excel file, named (i) Apect_Ratio_Tenocytes.xlsx; (ii) CAM_Series1&2.xlsx; and (iii) Seahorse_Raw Data_mean.xlsx, respectively.

### 3.1 Aspect ratio data

**File** Apect_Ratio_Tenocytes.xlsx

This excel file is open access published in Mendeley Data Aspect ratio data of rabbit Achilles tenocytes in vitro culture treated with Insulin-Like Growth Factor-1 (IGF-1), with Platelet-Derived Growth Factor-BB (PDGF-BB), or with both growth factors - Mendeley Data and contains 4 sheets, called *Control, IGF-1, PDGF-BB*, and *Mix*. All four sheets are structured the same and present data four time points: D0 = day 0 (starting point); D3 = day 3; D7 = day 7; and D14 = day 14 (all in line 3 of the sheet). For each of these time points, the columns are arranged and organized the same. In column A, there is the number of each cell that was measured in a consecutive manner. In column B, the width of the cell in µm is presented, followed by the length in µm in column C. After that, the aspect ratio is calculated in column D by width/length. In column E, the aspect ratio is calculated by length/width. The same is true for columns G-K; M-Q; and S-W, respectively.

### 3.2 CAM assay data

**File** CAM_Series1&2.xlsx

This excel file is open access published in Mendeley Data CAM assay data for chorioallantoic membrane treated with Insulin-Like Growth Factor-1 (IGF-1), with Platelet-Derived Growth Factor-BB (PDGF-BB), or with both growth factors - Mendeley Data and contains 6 sheets, called *Survival GraphPad; Junctions GraphPad; Vessel length GraphPad; Vessel_density_Sprouts_GrapPad; Vessel hierarchy GraphPad;* and *Angiogenic Index*, respectively.

**Fig. 1:**
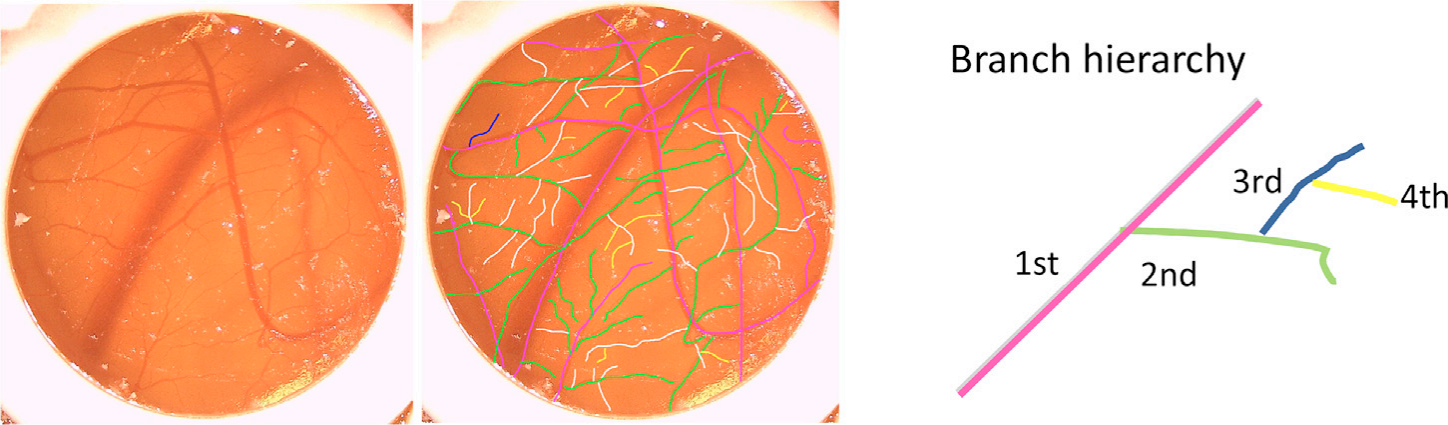
Scheme to show sprouts on the first level (pink), the second level (green), the third level (white/blue in the scheme on the left for better visibility) and the fourth level (yellow).

The first sheet *Survival GraphPad* shows the egg number in column A, the number of days survived in columns B and C, with single entries for all groups A-G in columns d-J, respectively. The second sheet entitled *Junctions GraphPad* includes the fold increase in junctions compared to starting point day 0, with group definition in column A, fold increase in junctions on day 2 in columns B-M, fold increase in junctions on day 4 in columns N-Y; and fold increase in junctions on day 7 in columns Z-AK, respectively. The third sheet called *Vessel lengths GraphPad* shows the same organization as the second sheet called *Junctions GraphPad;* however, here the values for the fold increase in average vessel length (lines 3–18) and in total vessel length (lines 24–41) is given. The next fourth sheet entitled *Vessel_density_Sprouts_GrapPad* is structured exactly the same as the previous third sheet. The only difference is that here the fold increase in vessel density data is shown (lines 3–18) and the fold increase in sprouts data (lines 24–41). After that, in sheet five entitled Vessel hierarchy GraphPad, fold increase in sprouts data are given for different hierarchical levels, with group A having sprouts on the first level, group B on the second level and group C and D on the third and fourth level, respectively (Fig. 1).

The sixth sheet named *Angiogenic Index* then includes the angiogenic activity index calculation, which is based on all previous readouts. Except for the data in sheet one on survival, all data from the rest of the sheets are shown again. What is new in this sheet is shown in columns N and M the calculated means and the corresponding angiogenic index. Furthermore, in column Q the mean overall angiogenic index per timepoint is denoted. In the subsequent columns R-W, one finds the single AAI values for junctions, average vessel length, total vessel length, vessel density, sprouts, and hierarchy, respectively. The same is done for day 2 (lines 1–85), day 4 (lines 98–164), and day 7 (lines 195–281).

### 3.3 Seahorse data on metabolism

**File** Seahorse_Raw Data_mean.xlsx

This excel file is open access published in Mendeley Data Seahorse-assessed metabolic data for rabbit adipose-derived stem cells and rabbit Achilles tenocytes treated with Insulin-Like Growth Factor-1 (IGF-1), with Platelet-Derived Growth Factor-BB (PDGF-BB), or with both growth factors simultaneously - Mendeley Data and contains 14 sheets, called *5A) Energy Map, 5B) OCR Tenos, 5C) ECAR Tenos, 5D) OCR ASC, 5E) ECAR ASC, 5F) ATP Prod. Tenos, 5* G*) ATP Prod. ASC, 5H) Basal res. ASC, 5I)* Max*. res. ASC, 5* J*) Spare res. ASC, 5* K*) Basal Glyc. Tenos, 5* L*) Induced Glyc Tenos, 5* M*) Glyc Tenos, and 5* L*) Glyc reserve*, respectively.

The first sheet named *5A) Energy Map* contains the group names in column A; and in columns B-G either the mitochondrial ATP production rate (lines 13–29) or the glycolytic ATP production rate (lines 33–50). In columns H and I, the mean and the standard deviation are calculated. In columns J and K, the mean and standard deviations are calculated under the omission of outliers that are colored in yellow in the list. The next four sheets called *5B) OCR Tenos, 5C) ECAR Tenos, 5D) OCR ASC, 5E) ECAR ASC* are all organized the same. They contain data on oxygen consumption rates (OCR) or extracellular acidification rates (ECAR) for tenocytes (Tenos) or adipose-derived stem cells (ASC), respectively. After that, in sheets *5F) ATP Prod. Tenos* and *5* G*) ATP Prod. ASC* the ATP production rate for tenocytes and for ASCs are given; with column A denoting the time in minutes and columns B-AK showing the values at the corresponding time point. The key for *RXY* is *XY* is the number of the rabbit donor that was used to perform the cell culture experiments. Column A in these two sheets represents the glycolytic, while column B shows the mitochondrial ATP production rate. Columns G-J calculate the means of these values. Finally, the rest 7 sheets, denoted as *5H) Basal res. ASC, 5I)* Max*. res. ASC, 5* J*) Spare res. ASC, 5* K*) Basal Glyc. Tenos, 5* L*) Induced Glyc Tenos, 5* M*) Glyc Tenos, and 5* L*) Glyc reserve* are all organized the same. In column A, the group is given. In column B, the well number of the wells in the plate are shown. In column C, the values in pmol/min/cells are presented. In column E finally the means are calculated.

## 4 Experimental Design, Materials and Methods

### 4.1 *Aspect ratio assessment of rabbit Achilles tenocytes* in vitro

To assess the impact of IGF-1, PDGF-BB or both GFs mixed on Achilles tenocytes isolated from rabbits, monolayer cell cultures were supplemented with these GFs, and photos were taken at distinct time points, according to the protocols published in the related research article [1].

### 4.2 *CAM assay experiments*

The CAM assay was performed according to a previously published protocol by dropping the GFs in different concentrations on the CAM surface [1]. Then, the angiogenic parameters were assessed as reported earlier [7]. We determined vessel density, number of junctions, total vessel length, mean vessel length, and branch hierarchy with four levels (Fig. 1), taking ImageJ software (version 2.9.0, NIH, Bethesda, MD, USA) for the analyses and to evaluate the photos of the CAM from days 0, 2, 4 and 7 for the timeline longitudinally [7].

### 4.3 Seahorse experiments

All Seahorse experiments to evaluate the metabolic responses of rabbit Achilles tenocytes and rabbit ASCs under GF supplementation were performed according to protocols that have been published in the related research article, utilizing a Seahorse XF-96 analyzer [1]. Briefly, the cells were seeded at 7500 cells/ well in 80 µL of growth medium. During the assays, the growth medium was exchanged with 200 µL of pre-warmed XF RPMI medium (pH 7.4, Agilent Technologies, 103,576–100) with 100 mM glucose, 1 mM pyruvate, and l-Glutamine for the mito stress test or without glucose for the glycolysis stress test. After equilibration, OCR (in pmoles per min) was measured to determine the rate of mitochondrial respiration, and ECAR (in mpH per min) was measured to assess lactate production or glycolysis.

## Limitations

The CAM assay was performed for 7 days and images were taken at 0, 2, 4 and 7 days. These experiments could be longer. Taking photos daily would enable a better interpretation because the dynamics of angiogenesis could be evaluated in more detail. Furthermore, Seahorse experiments are limited to only one concentration of the GFs. A dose-dependent analysis of the metabolic response would allow a more profound interpretation, which is necessary to elucidate the mechanistic aspects. To verify a proposed Warburg effect under PDGF-BB supplementation [8], additional HIF-1α data and their differential changes compared with the control group would allow verification of the underlying mechanism.

## Ethics Statement

The study was conducted in accordance with the veterinary office of the Canton Zurich, Switzerland, with the ethical approval under licence No 255/15.

## Data Availability

Mendeley DataSeahorse-assessed metabolic data for rabbit adipose-derived stem cells and rabbit Achilles tenocytes treated with Insulin-Like Growth Factor-1 (IGF-1), with Platelet-Derived Growth Factor-BB (PDGF-BB) (Original data). Mendeley DataCAM assay data for chorioallantoic membrane treated with Insulin-Like Growth Factor-1 (IGF-1), with Platelet-Derived Growth Factor-BB (PDGF-BB), or with both growth factors (Original data). Mendeley DataAspect ratio data of rabbit Achilles tenocytes in vitro culture treated with Insulin-Like Growth Factor-1 (IGF-1), with Platelet-Derived Growth Factor-BB (PDGF-BB), or with both growth factors (Original data).
